# Noncoding RNAs in human saliva as potential disease biomarkers

**DOI:** 10.3389/fgene.2015.00175

**Published:** 2015-05-07

**Authors:** Xianzhi Lin, Hsien-Chun Lo, David T. W. Wong, Xinshu Xiao

**Affiliations:** ^1^Department of Integrative Biology and Physiology, University of California, Los AngelesLos Angeles, CA, USA; ^2^Molecular Biology Institute, University of California, Los AngelesLos Angeles, CA, USA; ^3^School of Dentistry, University of California, Los AngelesLos Angeles, CA, USA; ^4^Jonnson Comprehensive Cancer Center, University of California, Los AngelesLos Angeles, CA, USA

**Keywords:** RNA-Seq, miRNA, piRNA, circular RNA, extracellular RNA, biomarker, saliva

Human saliva emerged as a research material in as early as the 17th century when investigators sought to understand the basis for salivary secretion (Garrett, 1975). Over the centuries, the focus of salivary research has evolved greatly and a wide range of topics has been examined (Garrett, 1975; Schipper et al., 2007). It is now known that the functions of saliva include at least lubrication, digestion of food, remineralization, prevention of demineralization, protection against microbial and viral infection, speech facilitation, and maintenance of oral and general health (Schipper et al., 2007; Malathi et al., 2014).

One active research area related to human saliva is the discovery of biomarker molecules for a variety of diseases. Compared to other body fluids, saliva is easily accessible in a non-invasive manner. However, it is also immediately exposed to the outside environment, thus may be confounded by a wide variety of environmental factors. Nevertheless, previous studies have established that at least some human molecules in saliva are highly stable and potential biomarkers have been examined for a number of oral and systemic diseases (Bonne and Wong, 2012; Schafer et al., 2014).

## Key components of human saliva

Saliva consists of both cellular and fluid contents. Epithelial cells, leukocytes, and erythrocytes are the three major human cell types, which co-exist with bacterial cells in human whole saliva (Aps et al., 2002). The fluid content of saliva is primarily generated by the salivary glands, but with additional contributions from blood, oral tissue, bacteria, viruses, and food remnants (Schipper et al., 2007). It mainly consists of water, macromolecules (such as glycoproteins, enzymes), small organic molecules, inorganic components (e.g., electrolytes), and metabolites from oral bacteria (Almstahl and Wikstrom, 2003; Aps and Martens, 2005; Schipper et al., 2007).

Many biomarker studies focused on profiling and quantification of proteins or RNA molecules in saliva. Since the diseases investigated for salivary biomarkers are often systemic, it is of great interest to identify circulating protein or RNA molecules that may have originated from disease-relevant cells (such as tumor cells). Such molecules reside outside of the cells in saliva and are often captured in cell-free saliva (CFS), the fraction of saliva with cellular contents removed (often by centrifugation). Most of the salivary RNAs appear to be highly degraded compared to full-length mRNAs in cellular compartments, possibly due to presence of RNA degradation enzymes in saliva and other body fluids (for circulating mRNAs) (Park et al., 2007). Notably, certain miRNA and mRNA molecules were shown to be highly stable, possible owing to protection by exosomes or protein complexes (Park et al., 2006, 2009; Palanisamy and Wong, 2010; Palanisamy et al., 2010).

## Technologies for salivary RNA profiling

About a decade ago, microarrays were applied to characterize the global profile of mRNAs in saliva (Li et al., 2004; Park et al., 2007). These studies revealed that there were over one thousand distinct mRNA molecules in human CFS (Li et al., 2004). In addition to mRNAs derived from coding genes, many noncoding RNAs (ncRNAs) were also detected. Data from these studies demonstrate that there are hundreds of microRNAs (miRNAs) in human saliva, and most of them likely exist in exosomes (Michael et al., 2010; Gallo et al., 2012).

However, microarray techniques have inherent limitations, such as the dependence on gene annotation and cross-hybridization noise. In recent years, more powerful techniques based on next generation sequencing (NGS) revealed additional coding and ncRNA species in human saliva (Spielmann et al., 2012; Bahn et al., 2015). In contrast to the hybridization-based microarrays, RNA sequencing (RNA-Seq) offers single nucleotide information, high sensitivity and accuracy in transcript detection, and the capability to detect novel RNA species and transcript isoforms (Lee et al., 2011, 2013; Li et al., 2012). An increasing number of bioinformatic tools are emerging for analysis of RNA-Seq data, ranging from rapid short read aligners to detailed examination of RNA expression patterns (Oshlack et al., 2010). Owing to these improvements, the catalog of human genes, especially ncRNA genes, has been greatly expanded (refer to Sai Lakshmi and Agrawal, 2008; Kozomara and Griffiths-Jones, 2014; Xie et al., 2014 for ncRNA databases).

## ncRNA molecules in saliva

In 2012, the Wong group reported the first global characterization of the human salivary transcriptome using high-throughput RNA-Seq (Spielmann et al., 2012). This study demonstrated that saliva harbors a wide variety of RNA species. More than 4000 distinct RNA molecules derived from coding or noncoding human RNAs were identified, including a small number of miRNAs. This study established that the RNA content in saliva is very diverse, which should be fully explored in future biomarker studies.

Recently, another in-depth analysis of human salivary extracellular ncRNA revealed novel insights regarding its RNA content and provided a comparative view of salivary ncRNAs relative to those of other body fluids (Bahn et al., 2015). Using human CFS, this study confirmed previous findings that miRNAs are stably and abundantly present in saliva (Patel et al., 2011), often harbored within exosomes (Gallo et al., 2012). miRNA expression profiles of healthy individuals were quantified and compared. Highly concordant miRNA expression was observed across individuals. Furthermore, considerable similarity was observed between miRNA expression levels of saliva and other body fluids (blood, cerebral spinal fluid (CSF)). Thus, these data suggest that salivary miRNAs could serve as candidate biomarkers, at least with equivalent promise as those derived from more invasive fluids.

A surprising observation from this study was the relative abundance of human piwi-interacting RNAs (piRNAs) in saliva. piRNAs are small ncRNAs typically ~26–32 nt in length observed in germ cells of both vertebrates and invertebrates (Aravin et al., 2006; Girard et al., 2006; Grivna et al., 2006; Lau et al., 2006; Watanabe et al., 2006; Das et al., 2008). piRNAs are known to target transposons and repress their mobility (Das et al., 2008; Malone and Hannon, 2009). The number of abundant piRNAs is less than that of miRNAs in saliva, despite the large number of annotated piRNAs in various databases (Sai Lakshmi and Agrawal, 2008; Bahn et al., 2015). Nevertheless, piRNA expression levels were highly concordant between healthy individuals, similarly as miRNA levels. However, in contrast to the consistent expression profile of miRNAs across body fluids, piRNAs were highly exclusive to saliva with very low abundance in blood or CSF. These observations indicate that salivary piRNAs may have originated from cells in the oral mucosa or salivary glands, rather than circulating from systemic organs via blood. Nevertheless, salivary piRNAs may impose systemic functional impact, which needs to be further investigated.

Another novel finding in this study was the discovery of circular RNAs (circRNAs) in CFS, which is the first report of the presence of circRNAs in an extracellular fluid (Bahn et al., 2015). CircRNAs were originally identified in RNA viruses (Sanger et al., 1976; Kos et al., 1986). Later, intracellular circRNAs generated from specific exons of coding genes were reported (Nigro et al., 1991; Cocquerelle et al., 1992; Capel et al., 1993). Recent studies demonstrated that circRNAs exist in many different cell types and species (Salzman et al., 2012, 2013; Jeck et al., 2013; Memczak et al., 2013). Some circRNAs are likely noncoding (Capel et al., 1993; Memczak et al., 2013; Guo et al., 2014), but others may code for proteins (Wang and Wang, 2015). The function of most circRNAs remains unknown. Two circRNAs were shown to function as miRNA sponges (Hansen et al., 2013; Memczak et al., 2013). However, this function may not apply to the majority of other circRNAs as they lack bioinformatic evidence of significant miRNA complementarity (Guo et al., 2014). The discovery of circRNAs in CFS indicates that this type of molecule may have extracellular function and should be considered as a type of candidate biomarker (Li et al., 2015).

Although mRNAs are highly degraded in saliva and other body fluids, small ncRNAs are often stable with reproducible expression across individuals. Indeed, miRNAs have been extensively studied in blood and other body fluids as potential disease biomarkers (Chen et al., 2008; Gilad et al., 2008; Mitchell et al., 2008; Wang et al., 2009, 2010; Fichtlscherer et al., 2010; Li et al., 2010; Liu et al., 2011). The similarity between miRNA profiles of saliva and other body fluids (Weber et al., 2010; Bahn et al., 2015) strongly supports the potential of using miRNAs (and possibly other ncRNAs) from human CFS as biomarkers for various human diseases.

## Salivary ncRNAs as potential biomarkers for diseases

Although at an early stage, salivary ncRNA studies have revealed potential disease biomarkers. Thus, far, most studies focused on miRNA expression in saliva. Table 1 summarizes a number of studies where miRNAs were assessed as putative biomarkers for oral squamous cell carcinoma (OSCC) (Park et al., 2009), parotid gland tumors (Matse et al., 2013), and esophageal cancer (Xie et al., 2013). In addition to oral and esophageal diseases, salivary ncRNAs were also examined as potential biomarkers for systemic diseases. In a clinical study focusing on Sjögren's Syndrome, a chronic autoimmune disease, the authors observed different miRNA expression patterns in minor salivary glands of Sjögren's Syndrome patient compared to healthy individuals (Alevizos et al., 2011). The disease group can be clearly distinguished from the normal group using the miRNA expression profile by principal components and hierarchical clustering analyses. A very recent study focused on pancreatic cancer, using samples of patients with pancreatic cancer, benign pancreatic tumor or healthy controls (Xie et al., 2015). The authors observed significant down-regulation of miR-3679-5p and up-regulation of miR-940 in the cancer group compared to the other groups (Table 1), suggesting salivary miRNA may potentially be used for early detection of pancreatic cancer.

**Table 1:** **Potential salivary miRNA biomarkers in cancer**.

**miRNA^a^**	**AUC^b^**	***p*-value**	**Up/Down-regulated^c^**	**Statistical test**
**HEALTHY CONTROL VS. ORAL SQUAMOUS CELL CARCINOMA (Park et al., 2009)**				
miR-200a	0.65	0.01	Down	Mann-Whitney U
miR-125a	0.62	0.03	Down	Mann-Whitney U
**BENIGN VS. MALIGNANT PAROTID GLAND TUMORS^d^ (Matse et al., 2013)**				
miR-132	0.9	0.003	Up	Wilcoxon 2-sided
miR-15b	0.9	0.0019	Up	Wilcoxon 2-sided
miR-140-5p	0.9	0.0003	Up	Wilcoxon 2-sided
miR-223	0.9	0.0542	Up	Wilcoxon 2-sided
**HEALTHY CONTROL VS. ESOPHAGEAL CANCER (Xie et al., 2013)**				
miR-10b-3p	0.702	0.013	Up (4.5)	Mann-Whitney U or Kruskall-Wallis H
miR-144	0.671	0.036	Up (6.7)	Mann-Whitney U or Kruskall-Wallis H
miR-21	0.698	0.015	Up (9.7)	Mann-Whitney U or Kruskall-Wallis H
miR-451	0.725	0.006	Up (5.4)	Mann-Whitney U or Kruskall-Wallis H
**HEALTHY CONTROL VS. RESECTABLE PANCREATIC CANCER (Xie et al., 2015)**				
miR-3679-5p	0.673	0.008	Down (2.162)	Mann-Whitney U
miR-940	0.68	0.006	Up (2.586)	Mann-Whitney U
**BENIGN PANCREATIC TUMORS VS. RESECTABLE PANCREATIC CANCER (Xie et al., 2015)**				
miR-3679-5p	0.716	0.007	Down (4.708)	Mann-Whitney U
miR-940	0.729	0.004	Up (3.02)	Mann-Whitney U
**HEALTHY CONTROL AND BENIGN PANCREATIC TUMORS VS. RESECTABLE PANCREATIC CANCER (Xie et al., 2015)**				
miR-3679-5p	0.688	0.002	Down (3.012)	Mann-Whitney U
miR-940	0.696	0.0001	Up (2.716)	Mann-Whitney U

a Validated by qRT-PCR.

b Area under Receiver Operating Characteristic (ROC) curve.

c Change in disease relative to control. The number in the parentheses represents the fold change.

d A combination of four miRNA exhibits discriminating power.

In addition to human ncRNAs, exogenous ncRNAs in saliva may also serve as potential disease biomarkers. The human-associated microbial communities have profound impact on the individual's physiological outcome (Human Microbiome Project Consortium, 2012). In human saliva, over 1500 bacteria have been identified and completely sequenced [Human Oral Microbiome Database; http://www.homd.org/]. Some studies have shown that saliva can be used to detect microbial infection (Schafer et al., 2014). In addition, both DNA and RNA viruses were detected in human saliva from viral infected hosts (Liou et al., 1992; Chen et al., 1997; Vieira et al., 1997; Shugars et al., 2001; Hermida et al., 2002; Mackiewicz et al., 2004; Goncalves et al., 2005; Balamane et al., 2010; Pride et al., 2012), thus could serve as biomarkers of viral infection. Thus, far, little is known regarding the landscape and function of exogenous ncRNAs in saliva.

## Future challenges and perspectives

A comprehensive ncRNA expression profile is emerging for human saliva including the presence of miRNAs, piRNAs, and circular RNAs (Ogawa et al., 2013; Bahn et al., 2015). More RNA species may be discovered in the future given the rapid evolution of new technologies and powerful bioinformatic methods. The value of saliva as a body fluid for biomarker discovery is just becoming widely recognized. However, there are a number of challenges in this field, most of which are general to usage of any body fluid in biomarker discoveries. One challenge lies in the unbiased isolation of short and long RNA molecules from saliva samples. Although this topic is under intensive investigation, improved methods that can retain most RNA species unbiasedly in an operator-independent manner are highly desired.

Another challenge is accurate quantification of ncRNA abundance, which is key to biomarker assessment. RNA yield from different samples may vary greatly, which calls for effective experimental and bioinformatic methods for normalization of RNA expression. Most RNA-Seq studies discussed above calculated RNA expression levels by normalizing the number of reads of a particular RNA molecule against the total number of mapped reads (i.e., the RPKM measure Mortazavi et al., 2008). However, to estimate the absolute concentration of an RNA molecule in a sample, synthetic spike-in RNAs with known concentration should be added to the RNA sample before library generation. This approach necessitates accurate measurement of RNA concentration of the sample and synthesis of a large number of spike-in RNAs with varying sequence contents and concentrations (see Williams et al., 2013 for a demonstration of this approach). This challenging approach, though highly desirable and necessary for clinical usage of a biomarker, has not been widely adopted.

A third major challenge is a better understanding of the biogenesis pathways of human ncRNAs in saliva, which constitutes the basis to assess whether and to what extent ncRNA expression can reflect a person's health condition. Salivary RNAs could be derived from systemic organs or local cells of the oral cavity. Cellular origins of candidate biomarkers for various diseases should be further examined to substantiate our understanding of the validity of the biomarkers. Indeed, the presence, origin, and functional roles of disease biomarkers are all essential questions general to studies of different types of biomarkers and diseases. A valid disease biomarker should be directly involved in disease mechanisms or indirectly associated/correlated with key pathways driving the pathogenesis of disease. The ultimate question is how knowledge gained in biomarker studies could be utilized to develop effective strategies for disease prevention and treatment, which closely relies on a clear understanding of disease mechanisms.

### Conflict of interest statement

David T. W. Wong is co-founder of RNAmeTRIX Inc., a molecular diagnostic company. He holds equity in RNAmeTRIX, and serves as a company Director and Scientific Advisor. The University of California also holds equity in RNAmeTRIX. Intellectual property that David Wong invented and which was patented by the University of California has been licensed to RNAmeTRIX. Additionally, he is a consultant to PeriRx. The other authors declare that the research was conducted in the absence of any commercial or financial relationships that could be construed as a potential conflict of interest.
